# Body weight following a multidomain lifestyle intervention in older adults at risk of dementia – A post-hoc analysis of the AgeWell.de-trial

**DOI:** 10.1186/s12877-026-08492-6

**Published:** 2026-10-15

**Authors:** Andrea E. Zülke, Adrian Schultz, Melanie Luppa, Solveig Weise, Alexander Bauer, Juliane Döhring, Carmen Wolf, Robert Philipp Kosilek, Jan Ziegler, Christian Brettschneider, Thomas Frese, Hanna Kaduszkiewicz, Jochen Gensichen, Wolfgang Hoffmann, Hans-Helmut König, Birgitt Wiese, David Czock, Jochen René Thyrian, Steffi G. Riedel-Heller

**Affiliations:** 1 Institute of Social Medicine, Occupational Health and Public Health (ISAP), University of Leipzig, Philipp Rosenthal Str. 55, 04103 Leipzig, Germany; 2 Institute of General Practice and Family Medicine, Martin-Luther- University Halle-Wittenberg, Magdeburger Str. 8, 06112 Halle, Saale, Germany; 3 Institute of General Practice, University of Kiel, Michaelisstr. 5, 24105 Kiel, Germany; 4 Institute of General Practice and Family Medicine, LMU University Hospital, LMU Munich, Nußbaumstr. 5, 80336 Munich, Germany; 5 Department of Health Economics and Health Service Research, University Medical Centre Hamburg-Eppendorf, Martinistr. 52, 20246 Hamburg, Germany; 6 German Center for Neurodegenerative Diseases (DZNE), site Rostock/Greifswald, Ellernholzstr. 1-2, 17489 Greifswald, Germany; 7 Institute for Community Medicine, University Medicine Greifswald (UMG), Ellernholzstr. 1-2, 17489 Greifswald, Germany; 8 MHH Information Technology – Science & Laboratory, Hannover Medical School, Carl-Neuberg Str. 1, 30625 Hannover, Germany; 9 Internal Medicine IX, Department of Clinical Pharmacology and Pharmacoepidemiology, Heidelberg University Hospital, Im Neuenheimer Feld 672, 69120 Heidelberg, Germany; 10 Faculty V: School of Life Sciences, University of Siegen, Am Eichenhang 50, 57076 Siegen, Germany

**Keywords:** Obesity, Weight loss, Primary prevention, Lifestyle, Healthy aging, Randomized controlled trial

## Abstract

**Background:**

Lifestyle interventions provide a promising approach to address higher body weight as a risk factor for dementia.

**Objective:**

The AgeWell.de-trial was primarily designed to preserve cognitive function and reduce dementia risk. In this post-hoc analysis, we investigated body weight following the multidomain intervention among older adults with overweight or obesity, as well as the role of motivation and confidence regarding weight loss.

**Methods:**

Analyses used data from the cluster-randomized AgeWell.de-trial, testing a multidomain intervention (dietary counselling, enhancement of physical, social and cognitive activity, medication review, intervention for cardiovascular risk factors, depressive symptoms/grief, if applicable) over 24 months in adults aged 60 to 77 years at risk for dementia. Associations between intervention allocation and body weight at follow-up were analyzed using linear regression, controlling for covariates, based on multiply imputed data.

**Results:**

The sample included *n* = 678 participants with a BMI ≥ 25 kg/m², mean age (SD): 68.9 (4.8), 52.1% female. Group allocation was not associated with body weight at 24-month follow-up (b (unstandardized regression coefficient) = -0.30, 95% CI: -0.51; 1.11). Older age (b = -0.14, 95% CI: -0.25; -0.03) and a high level of education (b = -1.28, 95% CI: -2.55; 0.00) were linked to lower body weight at follow-up, while higher baseline body weight predicted higher body weight at follow-up (b = 0.95, 95% CI: 0.91; 0.98). Levels of motivation and confidence regarding weight loss remained high but stable in both groups, regardless of group allocation.

**Conclusions:**

In this post-hoc analysis, group allocation was not associated with lower body weight at follow-up. Despite high levels of motivation and confidence regarding weight loss and favorable changes in diet and physical activity in the intervention group, these changes did not translate into measurable weight loss.

**Trial registration:**

AgeWell.de was prospectively registered in the German Clinical Trials Register on December 7th, 2017 (DRKS trial identifier DRKS00013555).

**Supplementary Information:**

The online version contains supplementary material available at https://doi.org/10.1186/s12877-026-08492-6.

## Introduction

The growing number of people living with dementia worldwide has resulted in an interest in modifiable risk factors and risk reduction to delay or prevent as many new cases of disease as possible. Current evidence suggests that up to 45% of dementia cases might be attributable to 14 potentially modifiable risk factors, i.e., low levels of education, hearing loss, visual loss, traumatic brain injury, arterial hypertension, excessive alcohol consumption, obesity, diabetes mellitus, elevated LDL-cholesterol, physical inactivity, depression, smoking, social isolation, and air pollution [1]. Lifestyle interventions, particularly multidomain interventions that address several risk factors simultaneously, e.g., diet, physical, cognitive and social activity, aim to reduce dementia risk and preserve cognitive function in middle-aged and older adults. Particularly, the beneficial effects of the Finnish Geriatric Intervention Study to Prevent Cognitive Impairment and Disability (FINGER) on cognitive function [2] and several other outcomes, including incident chronic morbidity [3], cerebrovascular events and validated dementia risk scores [4] have resulted in a global network (WW-FINGERS), aiming to tailor multidomain interventions to different local and socioeconomic contexts [5].

Overweight and obesity, commonly defined as a body mass index (BMI; body weight in kilograms divided by height in meters squared) of ≥ 25 kg/m² or ≥ 30 kg/m², have been identified as risk factors for cognitive impairment and dementia in systematic reviews and meta-analyses [6, 7]. This association might partly be mediated by other dementia risk factors like diabetes mellitus, hypertension or stroke, which are linked to overweight and obesity. In a large study using individual data from more than 1.3 million dementia-free individuals, higher BMI was linked to higher risk for dementia in long follow-ups (> 20 years), while lower risks for dementia were observed in short-term follow-ups [8]. It has been argued that this latter association, i.e., lower dementia risk in overweight/obese older adults, might be due to reverse causation, as (involuntary) weight loss frequently occurs during the preclinical dementia stage, making overweight and obesity seem protective against cognitive decline and dementia [9, 10]. In a meta-analysis of longitudinal cohorts and RCTs on weight loss in overweight and obese adults, intentional modest (2 kg) weight loss was linked to improvements in several cognitive domains at a median of 6 months follow-up [11].

Several (multidomain) lifestyle interventions aimed at preserving cognitive performance and reducing dementia risk have been conducted to date, with mixed results regarding primary outcomes [12–14]. Changes in risk factors for dementia have been suggested as promising surrogate outcomes, as effects on cognitive performance and incident dementia will likely require extensive intervention- and follow-up periods [4]. Positive effects of interventions targeting risk factors for dementia might also be beneficial for weight loss and management, e.g., by increasing physical activity, promoting a healthy diet, or strengthening self-regulatory skills more generally. Beyond that, lifestyle interventions are recommended not only for physical comorbidities of obesity, but also for mental health [15], as they provide low-risk, accessible possibilities for risk reduction.

While lifestyle changes can be challenging, especially in older age, a systematic review including 69 studies showed that lifestyle interventions had similar effects on weight loss and further relevant outcomes like physical function and cardiovascular parameters in older compared with younger adults [16]. Looking at lifestyle interventions targeting older adults at risk for dementia, the FINGER- and Healthy Aging through Internet Counselling in the Elderly (HATICE)-trials found beneficial effects of multidomain interventions on weight loss [2, 17]. A recent trial conducted in the US testing the Mediterranean-DASH Intervention for Neurodegenerative Delay (MIND)-diet found beneficial effects on cognitive performance in intervention group-participants who lost > 10% weight, however, between-group differences for weight loss at follow-up were non-significant [18, 19]. The Dutch Prevention of Dementia by Intensive Vascular Care (preDIVA)-trial found no significant effects on participants’ body weight [20]. The LookAHEAD-trial, targeting adults with type 2 diabetes in the US, found beneficial effects of a multidomain intervention on weight loss [21], further illustrating the potential for weight loss to play a role in dementia prevention.

Knowledge about potential predictors of intervention effects is crucial for design and implementation of lifestyle interventions. Currently, only few studies assess factors like motivation, self-efficacy, confidence or further potential determinants of successful behavior change, particularly weight loss, in older adults, as pointed out by respective reviews [22, 23]. Exceptions include analyses of the FINGER-trial, describing psychosocial determinants for intervention adherence [24], and studies using data from the AgeWell.de-trial, highlighting the role of self-efficacy for intervention adherence [25] and increased physical activity at follow-up [26]. Increasing knowledge on determinants of behavior change, particularly weight loss, is crucial to maximize effectiveness of lifestyle interventions and guide the implementation of strategies for dementia risk reduction into practice.

AgeWell.de was primarily designed to preserve cognitive function and reduce dementia risk, without body weight being a prespecified outcome. The present study represents a post-hoc analysis of body weight among participants with overweight or obesity. We examined whether group allocation was associated with body weight at 24-month follow-up among participants with overweight or obesity. Further, we examined associations of sociodemographic, lifestyle, psychosocial, and cardiometabolic characteristics with follow-up body weight; whether self-efficacy, motivation, and confidence regarding weight loss modified the association between group allocation and follow-up body weight; and whether motivation and confidence differed between groups at follow-up.

## Methods

### Study design and participants

The present study is a post-hoc secondary analysis of data from the 24-month cluster-randomized AgeWell.de trial. AgeWell.de was conducted at five study sites (Leipzig, Greifswald, Kiel, Munich, Halle) in Germany. Participants were aged 60 to 77 years, at risk for dementia, according to a Cardiovascular Risk Factors, Ageing and Incidence of Dementia (CAIDE; [27])-risk score of ≥ 9 points, and were recruited via general practitioner (GP) practices. GP practices (clusters) recruited participants after instruction by study personnel and were randomized in a 1:1-ratio either to a multidomain lifestyle intervention (intervention group; IG) including enhancement of physical, social and cognitive activity, dietary counselling and medication review, intervention targeting depressive symptoms or grief following bereavement, if applicable. If cardiovascular risk factors for dementia, e.g. smoking or overweight/obesity were present, participants were provided information on how to reduce or eliminate said risk factors (detailed description below) or general health advice (control group; CG). Primary outcome was cognitive performance at 24-months follow up. Secondary outcomes included (instrumental) activities of daily living, social inclusion, (health-related) quality of life, and depressive symptoms. Baseline assessments were conducted between 07/2018 and 10/2019 (n _baseline_: 1,030; mean age in years (SD): 69.0 (4.9)), follow-up assessments took place between 07/2020 and 01/2022 (n _follow−up_: 819). Trained study nurses conducted baseline and follow-up examinations and instruction of intervention components using structured interviews at participants’ homes. Study design [28], baseline sample characteristics [29] and main intervention effects [30, 31] are described in detail elsewhere. Reporting of the present study follows the Consolidated Standards of Reporting Trials (CONSORT) statement and its extension for cluster randomized trials.

### Intervention components

For enhancement of *physical activity*, IG participants conducted standardized exercises for strength and flexibility/balance at their homes twice a week and set individual goals for aerobic exercises to be conducted 3–5 times/week, based on individual preferences. Participants further received a pedometer to track the number of walked steps on a daily basis. Individual goals for enhancement of *social activity* were set with each participant, based on personal preferences, aiming to engage in ≥ 1 social activity per week.

Participants received tablet computers with the cognitive training software NeuroNation © for enhancement of *cognitive activity*, to be used three times a week for ≥ 15 min; furthermore, individual goals for cognitively stimulating activities were scheduled, based on participants’ preferences.

Regarding *dietary counselling*, participants received advice (oral and written information) based on guidelines of the German Nutrition Society (DGE), recommending, e.g., consumption of ≥ 5 portions of fruit and vegetables daily, limiting intake of salt and sugar, preferring wholegrain over other grain products and consumption of unsweetened beverages.

For the *medication review*, information on current medication was obtained from attending GPs and participants alike. Based on information on prescribed and actual medication, an electronic evaluation was conducted at the expertise center for clinical pharmacology and pharmacoepidemiology at University Hospital Heidelberg to identify anticholinergic drugs, potentially missing medication for cardiovascular diseases or diabetes mellitus, potentially serious drug-drug interactions, and potential contraindications due to renal impairment. Based on said examination, standardized recommendations were sent per mail to attending GPs, including suggestions for modification of participants’ medication, if applicable.

In case of further *cardiovascular risk factors* for dementia, i.e., *smoking* and *overweight/obesity*, study nurses provided oral and written information on the respective risk factors and suggested ways to reduce said risk factors (e.g., smoking cessation).

In case of *depressive symptoms* and *grief after bereavement*, participants were encouraged to contact their attending GP for support. Participants were further provided with information on depression, grief reactions, and received addresses of local self-help groups and help lines to be contacted if needed.

Following instructions by study nurses after the baseline interview, participants conducted the intervention independently. Five telephone sessions between study nurses and participants were scheduled during the course of the intervention (2, 4, 8, 16, and 20 months after baseline) to ensure adherence to the intervention and keep up motivation. During the same intervention period (24 months), the control group (CG) received written information on lifestyle and dementia risk and GP treatment as usual (GPTAU).

### Outcomes and covariates

The present study focused on AgeWell.de participants who were overweight or obese, defined by a BMI ≥ 25.0 and < 30.0 kg/m² or ≥ 30.0 kg/m², respectively. Body weight was assessed using body scales during an anthropometric examination during the baseline and follow-up visits conducted by trained study nurses, assessing participants’ weight, height and blood pressure. For the present post-hoc analysis, body weight at 24-month follow-up was examined as the outcome. BMI was used to define the analysis sample according to established cut-offs for overweight and obesity. Only participants who were overweight or obese received respective information on associations between overweight/obesity and dementia risk and were encouraged to lose weight in course of the AgeWell.de intervention.

Participants provided information on *sociodemographic factors*, i.e., sex, age, and level of education, during the baseline interview. Level of education was assessed according to the Comparative Analysis of Social Mobility in Industrial Nations (CASMIN)-classification [32], including information on vocational and professional education, resulting in three categories: low, intermediate, and high.

Participants’ *diet* was assessed using a validated food frequency questionnaire (FFQ; [33]). The FFQ captured frequency and amount of 53 foods/beverages consumed during the past four weeks (frequency categories: never; once a month; two to three times a month; one to two times a week; three to four times a week; five to six times a week; one time per day; two times per day; three times per day; four to five times per day; more than five times per day). As described in previous studies [34, 35], a sum score for a healthy diet was calculated, following an approach by Voortman and colleagues [36]. This sum score, based on the Dutch dietary guidelines, included 11 components of a healthy diet (consumption of fruit and vegetables; whole-grain products; legumes; nuts; fish; tea; dairy products; red/processed meat; sugar-containing beverages; alcohol). One point was awarded for each respective item that was fulfilled, resulting in a total score ranging from 0 to 11. Content and recommendations of the Dutch and German guidelines for healthy nutrition are highly comparable [37]. However, the former were designed based on systematic reviews and meta-analyses and aimed to provide recommendations contributing to lower risk of several chronic diseases [36]. Higher adherence to the Dutch dietary guidelines has been associated with lower risk for depression and stroke, larger total brain volume, gray and white matter volume, and hippocampal volume [38].

*Physical activity* was assessed using a standardized questionnaire based on Luck and colleagues [39]. Questions assessed the frequency of the following activities: cycling; hiking/long walks; swimming; gymnastics; fitness/strength training; further sports; using a pedometer; gardening; bowling, dancing, or further physical activities; answering options: never (0 points); less than once a week (1 point); once a week (2 points); several times a week (3 points); every day (4 points). For each individual, the points for all activities were summed up to calculate a mean activity score (range: 0–36), where higher values indicate higher levels of physical activity.

*Self-efficacy* was assessed using the Generalized Self-Efficacy Scale (GSE; [40]). The GSE consists of ten items (e.g. “It is easy for me to stick to my aims and accomplish my goals.”), scored on a 4-point Likert-scale, ranging from 0 (not at all true) to 3 (exactly true). Items were summed up for each participant to derive the total score as a measure of self-efficacy, with higher values indicating higher levels of self-efficacy.

Participants‘ *motivation* and *confidence* in their ability for weight loss were assessed using a single-item question, respectively („How important is it for you to lose weight?“; “How confident are you that you can lose weight?”), answering options: not at all (1 point), rather little (2 point), somewhat (3 points), rather a lot (4 points), very much (5 points).

*Cardiovascular and metabolic conditions* associated with overweight and obesity (hyperlipidemia; hypertension; diabetes mellitus; cardiac insufficiency; cardiac arrhythmia) were assessed as diagnoses given by the attending GP (0 = no, 1 = yes).

Participants provided information on *smoking status*, answering the question: “Do you currently smoke, even if only occasionally?” (0 = no, 1 = yes).

### Statistical analyses

Sample characteristics at baseline are described as means (M) and standard deviations (SDs), or percentages. Follow-up body weight was analyzed using multivariable linear regression with baseline body weight included as a covariate to account for baseline differences in body weight and to counteract regression to the mean [41]. Regression coefficients represent adjusted differences in body weight at 24-month follow-up. The model included group allocation, age, sex, level of education, marital status, self-efficacy, motivation and confidence regarding weight loss, smoking, physical activity, dietary quality, diabetes, hypertension, hyperlipidemia, cardiac insufficiency, and cardiac arrhythmia. For sensitivity analyses examining whether associations were robust when conceptually related covariates were considered jointly, domain-specific composite scores for *lifestyle*, *psychosocial factors* and *cardiometabolic conditions* were calculated by standardizing the individual variables (z-scores) and averaging the standardized values within each domain. The lifestyle score comprised smoking, physical inactivity and unhealthy diet; the psychosocial score comprised self-efficacy, motivation and confidence regarding weight loss; the cardiometabolic score comprised diabetes, hypertension, hyperlipidemia, cardiac insufficiency and cardiac arrhythmia.

Continuous covariates were included in their original metric, while categorical covariates were entered as indicator variables. Potential non-linearity in the association between age and follow-up body weight was assessed by additionally including quadratic age term in the regression model. Clustering of participants in GP practices was accounted for using robust sandwich estimators. The observed ICC indicated meaningful clustering at GP level, supporting the use of cluster-robust estimation. The alpha-level (two-tailed) was set at 0.05. To assess multicollinearity, variance inflation factors were calculated. Respective values ranged between 1.02 and 1.67 (mean VIF: 1.21, not tabulated; values ≥ 10 commonly regarded as critical [42]), arguing against problematic levels of multicollinearity between predictors.

Missing covariate data were addressed using multiple imputation by chained equations, following recommendations for handling of missing data in clinical trials [43]. As no indication of systematic item missingness was detected (please see Sample description for details), we assumed no critical violation of the missing-at-random-assumption. BMI and body weight were not imputed. Twenty imputed datasets were generated after five burn-in iterations. Predictive mean matching with 10 nearest neighbors was used for continuous variables, ordered logistic regression for ordinal variables, and logistic regression for binary variables. Predictors included intervention group, age, sex, education, marital status, baseline and follow-up body weight, baseline BMI and cognitive performance. Food-frequency questionnaire items were imputed at item level, and the dietary components and total score were recalculated within each dataset; estimates were pooled according to Rubin’s rules. Regression results are based on pooled sets of all 20 imputed datasets. To ensure robustness of our findings, sensitivity analyses were conducted using only observations with no missing data on any covariate (complete case-analysis).

Changes in motivation for weight loss and confidence in one’s ability to lose weight were assessed visually, using alluvial plots [44], as well as by ordered logistic regression analyses with cluster-robust sandwich estimators, presented as odds ratios and 95% CIs. Descriptive distributions and alluvial plots of motivation and confidence were based on observed data. Baseline-adjusted between-group differences at follow-up were examined using ordered logistic regression with cluster-robust standard errors, using imputed data; results are presented as odds ratios and 95% confidence intervals.

No formal adjustment for multiple testing was applied. Considering the exploratory nature of our analyses, findings from individual covariates, subgroup analyses, and interaction tests were interpreted with appropriate caution. All analyses were conducted using Stata (SE) 16.0.

## Results

### Sample description

Of *n* = 819 participants who attended the follow-up assessment, 777 provided data on body weight at both timepoints. As the current study focused on overweight/obese adults, observations with a BMI < 25 kg/m² at baseline were excluded (*n* = 99), resulting in an analysis sample of *n* = 678. Older participants were more likely to be missing information on diabetes (*p* = .012), motivation for weight loss (*p* = .019) and self-efficacy (*p* = .005). Men were more likely to have missing information on motivation (*p* = .025) and confidence (*p* = .002) regarding weight loss. A low level of education was linked to higher probability of missing values for diet (*p* = .002), while probability of missing data in any of the other included covariates was not linked to level of education. Item-level missingness was not linked to group allocation for any covariate. Baseline sample characteristics are described in Table 1. On average, participants had a body weight of 93.9 kg (SD: 15.8) and a BMI of 32.7 kg/m² (SD: 4.6). At baseline, 50.0% of CG participants and 44.5% of IG participants selected the highest motivation category.

**Table 1:** Sample characteristics of AgeWell.de participants with a BMI ≥ 25 kg/m² at baseline

Variable	Total sample (*n* = 678)	Intervention group (*n* = 307)	Control group (*n* = 371)	Missing, *n* (%)
*Sociodemographic information*				
Age, M (SD)	68.9 (4.8)	69.1 (4.8)	68.7 (4.7)	
Sex				
Male, n (%)	325 (47.9)	153 (49.8)	172 (46.4)	
Female, n (%)	353 (52.1)	154 (50.2)	199 (53.6)	
Married/in a partnership, n (%)	450 (66.4)	207 (67.4)	243 (65.5)	
Education^1^				
Low, n (%)	150 (22.1)	77 (25.1)	73 (19.7)	
Intermediate, n (%)	370 (54.6)	163 (53.1)	207 (55.8)	
High, n (%)	158 (23.3)	67 (21.8)	91 (24.5)	
*Lifestyle factors*				
Body weight in kg, M (SD)	92.3 (15.9)	92.5 (14.4)	92.1 (17.1)	
Body Mass Index in kg/m², M (SD)	32.1 (4.7)	32.0 (4.4)	32.1 (5.0)	
Healthy diet score, M (SD)	4.2 (1.7)	4.2 (1.6)	4.2 (1.7)	119 (17.8)
Physical activity score, M (SD)	9.9 (4.3)	9.9 (4.5)	9.9 (4.1)	22 (0.3)
*Cardiovascular/metabolic risk factors*				
Hyperlipidemia, n (%)	486 (71.7)	218 (71.0)	268 (72.2)	11 (1.6)
Diabetes mellitus, n (%)	286 (42.2)	122 (39.7)	164 (44.2)	6 (0.9)
Smoking, n (%)	68 (10.0)	34 (11.1)	34 (9.2)	8 (1.2)
Cardiac insufficiency, n (%)	65 (9.6)	29 (9.5)	36 (9.7)	19 (2.8)
Cardiac arrhythmia, n (%)	99 (14.6)	48 (15.6)	51 (14.8)	9 (1.3)
*Psychosocial factors*				
Self-efficacy, M (SD)	22.6 (4.8)	22.8 (4.7)	22.4 (4.9)	23 (3.4)
Motivation for weight loss, M (SD)	3.9 (1.2)	3.8 (1.3)	4.0 (1.2)	73 (10.8)
Motivation for weight loss, level				
Not at all, n (%)	44 (7.3)	24 (8.5)	20 (6.2)	
Rather little, n (%)	17 (2.8)	6 (2.1)	11 (3.4)	
Somewhat, n (%)	172 (28.4)	95 (33.8)	77 (23.8)	
Rather a lot, n (%)	85 (14.1)	31 (11.0)	54 (16.7)	
Very much, n (%)	287 (47.4)	125 (44.5)	162 (50.0)	
Confidence in weight loss, M (SD)	3.3 (1.2)	3.3 (1.2)	3.3 (1.2)	79 (11.7)
Confidence in weight loss, level				
Not at all, n (%)	56 (9.0)	25 (9.0)	31 (9.7)	
Rather little, n (%)	40 (6.7)	14 (5.0)	26 (8.1)	
Somewhat, n (%)	295 (49.3)	150 (54.0)	145 (45.2)	
Rather a lot, n (%)	81 (13.5)	34 (12.2)	47 (14.6)	
Very much, n (%)	127 (21.2)	55 (19.8)	72 (22.4)	

^1^ assessed according to CASMIN: Comparative Analysis of Social Mobility in Industrial Nations

### Associations with body weight at follow-up

On average, body weight increased by 1.3 kg (SD: 5.0) in the total sample between baseline and follow-up (IG: 1.1, SD: 5.1; CG: 1.4, SD: 5.0), without adjusting for baseline-body weight and further covariates (not tabulated). Results of the linear regression analyses, investigating the association between group allocation and body weight at follow-up, are described in Table 2. Group allocation was not associated with body weight at follow-up. Higher age and a high level of education were linked to lower body weight at follow-up. Body weight showed strong tracking over time, i.e., higher baseline body weight was associated with higher body weight at follow-up after adjustment for the remaining covariates. No further covariates were associated with group differences in body weight at follow-up. Neither self-efficacy, motivation for weight loss, nor confidence in one’s ability to lose weight were significantly associated with weight differences at follow-up. Results remained unchanged when using domain-specific composite scores instead of individual covariates, with no significant effect of respective scores on body weight at follow-up (lifestyle score: smoking, physical activity, diet; b = -0.08; 95% CI: -0.70; 0.54; psychosocial score: motivation, confidence, self-efficacy; b = 0.003, 95% CI: -0.47; 0.48; cardio-metabolic score: diabetes mellitus, hypertension, hyperlipidemia, cardiac insufficiency, cardiac arrhythmia; b = -0.76, 95% CI: -1.48; 0.00; not tabulated).

**Table 2:** Adjusted associations with body weight at 24-month follow-up among participants with BMI ≥ 25 kg/m² (*n* = 678; multiply imputed data)

Variable	b (b)	95% CI	*p*
Intervention group (ref.: control group)	0.30	-0.51; 1.11	0.458
Body weight at baseline in kg, M (SD)	0.95	0.91; 0.98	**< 0.001**
Age (years)	-0.14	-0.25; -0.03	**0.016**
Female sex (ref.: male)	-0.87	-1.82; 0.08	0.074
Married/relationship (ref.: single)	-0.72	-1.63; 0.20	0.123
Education level intermediate (ref.: low)	-0.00	-1.09; 1.08	0.995
Education level high (ref.: low)	-1.28	-2.55; 0.00	**0.049**
Self-efficacy (GSE-score)	-0.05	-0.14; 0.04	0.265
Motivation for weight loss	-0.02	-0.40; 0.35	0.911
Confidence in ability for weight loss	0.19	-0.16; 0.54	0.278
Smoking (ref.: non-smoker)	-0.20	-1.44; 1.03	0.745
Physical activity, total score	0.01	-0.08; 0.09	0.903
Healthy diet, total score	0.01	-0.25; 0.26	0.952
Diabetes mellitus	-0.45	-1.11; 0.20	0.171
Hypertension	-0.39	-1.44; 0.66	0.466
Hyperlipidemia	-0.39	-1.30; 0.52	0.397
Cardiac insufficiency	-1.02	-2.73; 0.70	0.243
Cardiac arrhythmia	0.31	-0.80; 1.42	0.578

Regression results based on multiply imputed data; b: coefficient; CI: confidence interval; GSE: Generalized Self-Efficacy Scale; ref.: reference

**Fig. 1:**
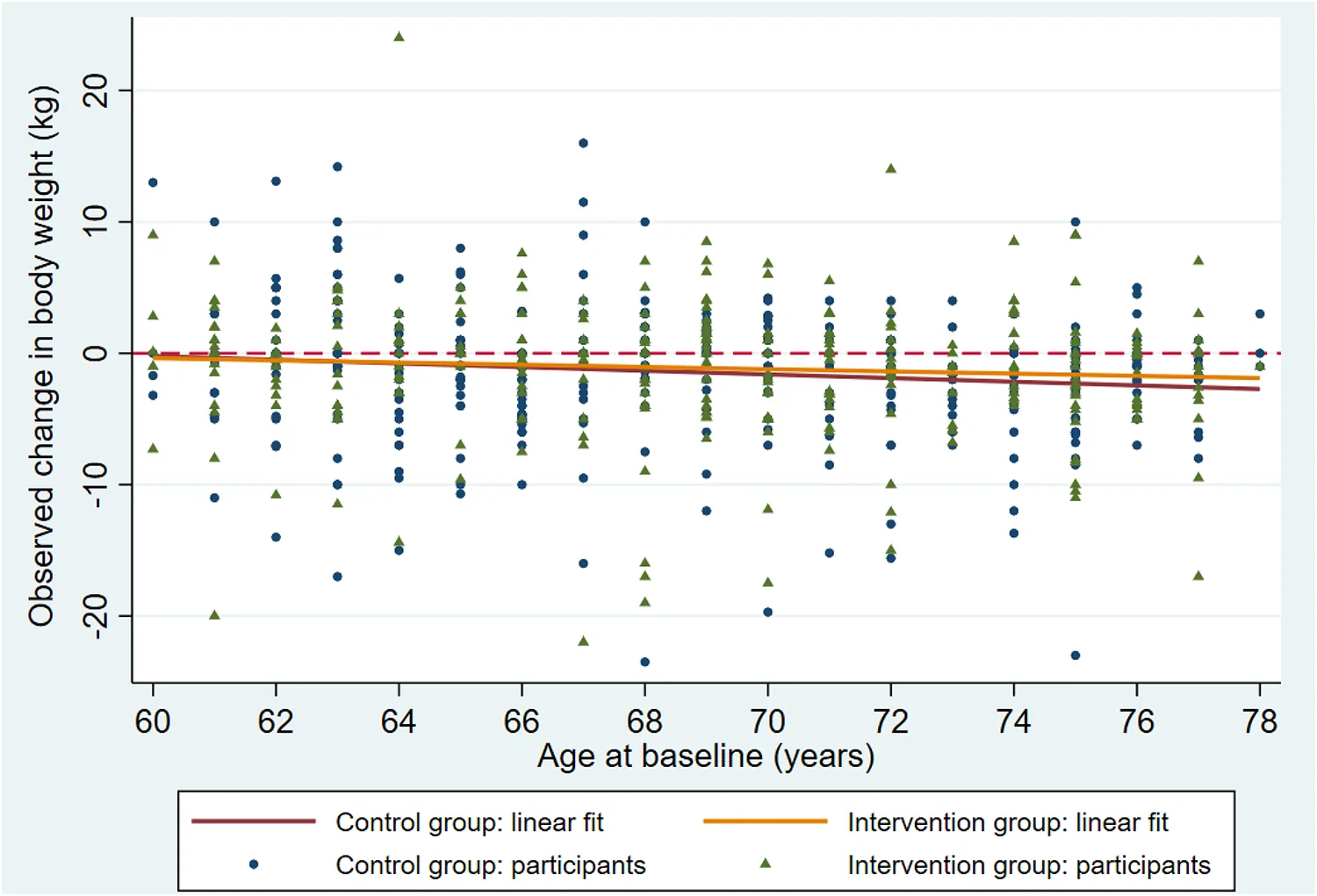
Observed body weight change from baseline to 24-month follow-up by baseline age and study group. Points represent individual participants, solid lines represent unadjusted group-specific linear fits. Negative values indicate weight loss; the dashed horizontal line indicates no weight change

To test potential non-linearity in the association between age and follow-up body weight, the model was additionally estimated including a quadratic age term. No evidence of a non-linear association was detected (b _age²_= 0.0003, 95% CI: -0.02; 0.02; *p* = .973), therefore, age was retained as a linear predictor. Figure 1 descriptively displays observed body weight change from baseline to follow-up, according to age and group allocation.

Further analyses investigated potential interaction effects of group allocation and self-efficacy, motivation and confidence regarding weight loss on body weight at follow-up. None of the respective interaction terms were significant (b _self−efficacy*IG_ = 0.00, 95% CI: -0.18; 0.18; *p* = .998; b _motivation*IG_ = 0.43, 95% CI: -0.28; 1.13; *p* = .235; b _confidence*IG_ = -0.13, 95% CI: -0.81; 0.56; *p* = .713; see Supplementary Table 1).

In sensitivity analyses using only cases with complete data (*n* = 557), highly similar results were observed. Again, higher age was linked to lower body weight at follow-up (b = -0.21, 95% CI: -0.36; -0.08; *p* = .002), as was a high level of education (b = -2.13, 95% CI: -3.60; -0.65; *p* = .005) and being married/in a relationship (b = -1.17, 95% CI: -2.12; -0.21; *p* = .018). Higher body weight at baseline was linked to higher body weight at follow-up (b = 0.94, 95% CI: 0.90; 0.98; *p* < .001), see Supplementary Table 2). Potential interaction effects of group allocation and self-efficacy, motivation and confidence regarding weight loss on body weight at follow-up were non-significant also in complete case-analyses (b _self−efficacy*IG_ = 0.29, 95% CI: -1.52; 2.10; b _motivation*IG_ = 0.64, 95% CI: -0.26; 1.54; b _confidence*IG_ = -0.57, 95% CI: -1.43; 0.28; not tabulated).

### Subgroup analyses

Following the main analyses, we examined associations between group allocation and body weight at follow-up in the subgroup of participants with obesity (BMI ≥ 30 kg/m²; *n* = 428, IG / CG: 232 / 196; mean BMI (SD): 34.7 kg/m² (3.9)). Results are described in Table 3. Again, group allocation was not linked to participants’ body weight at follow-up. Except for body weight at baseline, no further sociodemographic, nor health-related, lifestyle- or psychosocial covariates were linked to differences in body weight at follow-up. Again, we investigated potential interactions between self-efficacy, motivation for weight loss and confidence in ability to lose weight and group allocation, revealing non-significant associations with body weight at follow-up (b _self−efficacy*IG_ = 0.05; 95% CI: -0.15; 0.26; b _motivation*IG_ = 0.88, 95% CI: -0.22; 1.97; b _confidence*IG_ = -0.40; 95% CI: -1.34; 0.56; not tabulated).

**Table 3:** Adjusted associations with body weight at follow-up in participants with obesity (BMI ≥ 30 kg/m²; *n* = 428), imputed data

Variable	b (b)	95% CI	*p*
Intervention group (ref.: control group)	0.18	-0.98; 1.34	0.758
Body weight at baseline in kg, M (SD)	0.93	0.88; 0.99	**< 0.001**
Age (years)	-0.13	-0.28; 0.20	0.089
Female sex (ref.: male)	-1.21	-2.67; 0.26	0.105
Married/relationship (ref.: single)	-0.74	-1.94; 0.47	0.227
Education level intermediate (ref.: low)	0.17	-1.32; 1.67	0.821
Education level high (ref.: low)	-1.03	-2.84; 0.78	0.261
Self-efficacy (GSE-score)	-0.07	-0.18; 0.04	0.201
Motivation for weight loss	0.19	-0.42; 0.79	0.543
Confidence in ability for weight loss	0.17	-0.29; 0.64	0.463
Smoking (ref.: non-smoker)	0.08	-1.73; 1.89	0.930
Physical activity, total score	-0.00	-0.14; 0.13	0.957
Healthy diet, total score	0.05	-0.32; 0.41	0.800
Diabetes mellitus	-0.36	-1.25; 0.54	0.432
Hypertension	-0.72	-2.24; 0.80	0.348
Hyperlipidemia	-0.16	-1.34; 1.02	0.790
Cardiac insufficiency	-1.20	-3.56; 1.17	0.317
Cardiac arrhythmia	0.61	-1.07; 2.29	0.471

b: coefficient; CI: confidence interval; GSE: Generalized Self-Efficacy Scale; ref.: reference

Sensitivity analyses using complete data (*n* = 386) revealed highly comparable results, the only exception being an association between diagnosed cardiac arrhythmia and higher body weight at follow-up in participants with obesity (b = 1.81, 95% CI: 0.10; 3.52; see Supplementary Table 3).

### Changes in motivation and confidence regarding weight loss

**Fig. 2:**
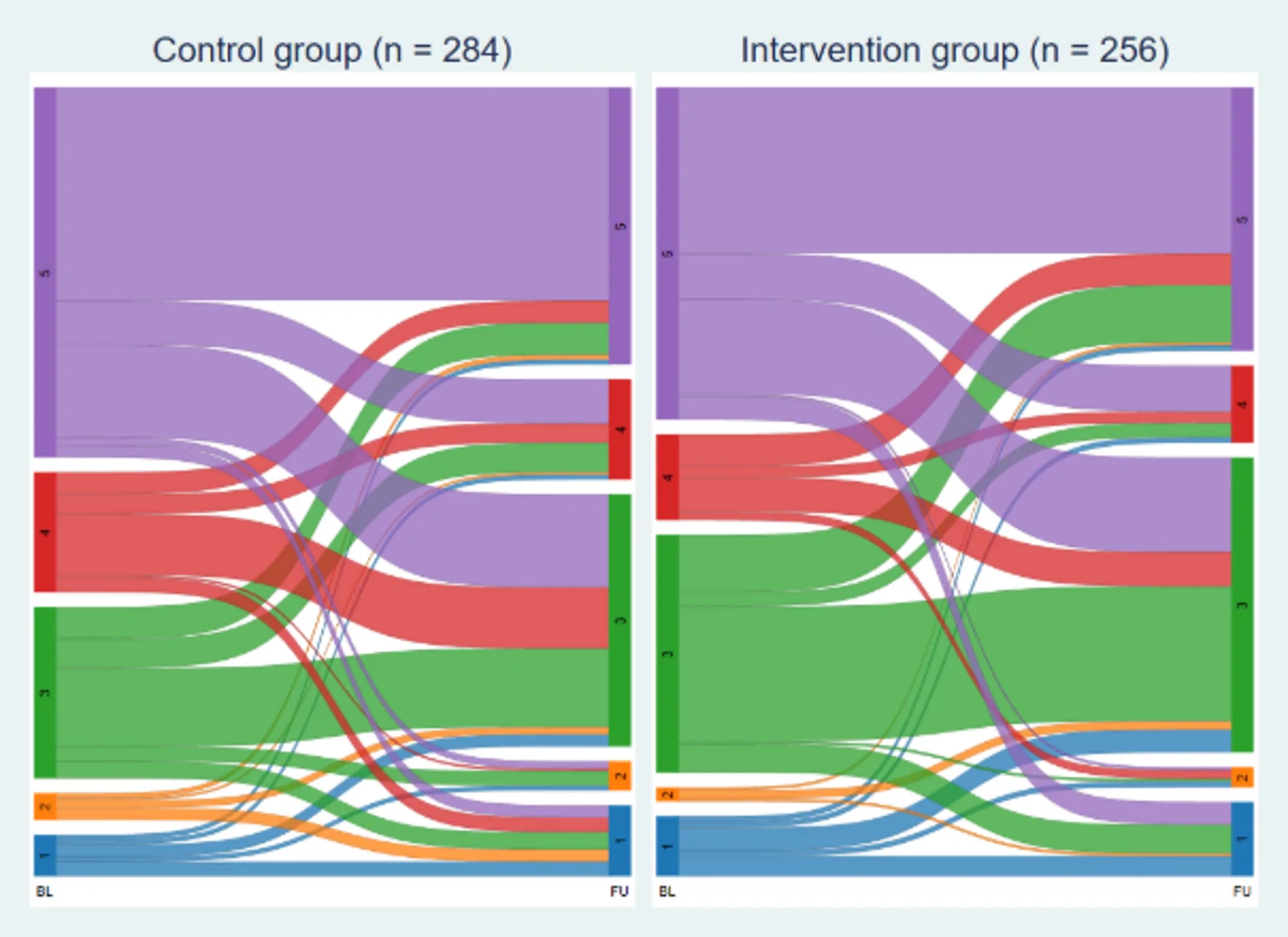
Changes in motivation for weight loss, by group allocation. Alluvial plot showing transitions in participants’ ratings of motivation for losing weight from baseline to follow-up, by group allocation. Response categories range from 1 (“not at all important”; blue) to 5 (“very much”; purple). The width of each flow represents the number of participants moving between categories across time points. BL: baseline; FU: follow-up

**Fig. 3:**
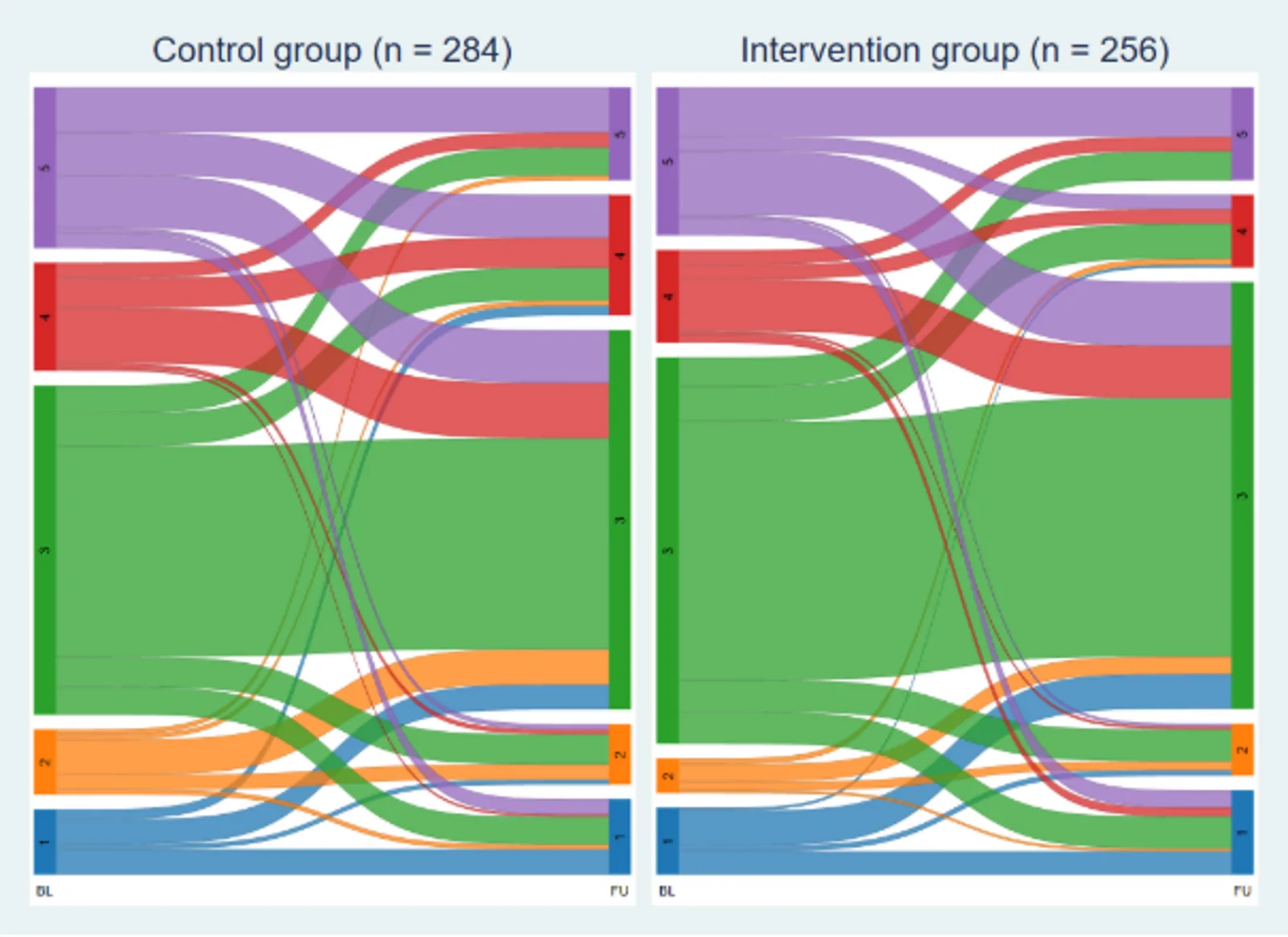
Changes in confidence in ability to lose weight, by group allocation. Alluvial plot showing transitions in participants’ ratings of confidence in their ability to lose weight from baseline to follow-up, by group allocation. Response categories range from 1 (“not at all”; blue) to 5 (“very much”; purple). The width of each flow represents the number of participants moving between categories across time points. BL: baseline; FU: follow-up

Figures 2 and 3 illustrate changes in motivation and confidence regarding weight loss between baseline and follow-up descriptively by group allocation, using un-imputed data.

Descriptively, responses to the question “How important is it for you to lose weight?” remained largely stable over time, with the largest flows remaining within the same response category, particularly in the middle and higher categories. No visually meaningful differences in transition patterns were observed between groups (Fig. 2). Applying ordered logistic regression analyses using imputed data, changes in stages of motivation for weight loss did not differ between groups when controlling for baseline-values of motivation for weight loss, age, sex, education and marital status, (OR = 1.07, 95% CI: 0.78; 1.46; *p* = .694).

Similar to the findings for motivation, responses to the question “How confident are you that you can lose weight?” were largely stable over time in descriptive analyses, with the majority of participants remaining in the same response category, particularly in the middle range of the scale (Fig. 3). Transition patterns appeared highly comparable between groups, and ordered logistic regression analyses using imputed data confirmed no significant between-group differences in change after adjusting for baseline values, age, sex, education and marital status (OR = 0.84, 95% CI: 0.61; 1.16, *p* = .289).

To investigate behavioral factors potentially linked to weight loss and motivation/confidence regarding weight loss, changes in the healthy diet- and physical activity-scores were analyzed using linear regression analyses. Based on imputed data and controlling for baseline values of the respective scores, age, sex, education and marital status, beneficial changes in the IG were detected for adherence to a healthy diet (b = 0.24, 95% CI: 0.01, 0.46; *p* = .039) and physical activity (b = 2.22, 95% CI: 1.56; 2.87; *p* < .001; Supplementary Table 4).

## Discussion

In this post-hoc analysis of the AgeWell.de trial, we investigated body weight following a multidomain lifestyle intervention in older adults at risk for dementia who were overweight or obese. AgeWell.de was primarily designed to preserve cognitive function and reduce dementia risk rather than to reduce weight. Controlling for covariates, no association between group allocation and body weight was detected either for participants with overweight or obesity. Older participants and those with a high level of education had lower body weight at follow-up, while higher baseline body weight predicted higher body weight at follow-up. Neither self-efficacy, nor motivation or confidence regarding weight loss, or further sociodemographic, health-related, lifestyle- or psychosocial covariates were linked to group differences in body weight at follow-up.

Our results differ from previous studies testing lifestyle interventions for dementia risk reduction, i.e., the FINGER- and HATICE-trials, reporting beneficial effects of interventions delivered in-person (FINGER) or remotely (HATICE) on weight loss [2, 17]. However, in a comprehensive analysis of several multidomain intervention trials targeting cognitive decline/dementia risk, BMI/obesity was way less responsive to change due to the intervention than other risk factors for dementia, e.g., diet or hypertension [4]. Respective differences might partly be due to variations in intervention content, dose and intensity, as well as sampling of participants. Future studies might benefit from more standardized assessments of adherence and lifestyle changes to facilitate comparison of different interventions [45]. Our findings indicate that multidomain lifestyle interventions targeting dementia risk/cognitive decline do not necessarily translate into weight changes, especially if weight loss is not an explicit intervention outcome. Several trials explicitly focusing on weight loss in older adults were able to induce significant changes in body weight, e.g., the LookAHEAD-trial, testing a multidomain intervention in older adults with pre-diabetes over an 8-year intervention period [21], or the IDEA trial, applying an 18-month diet + exercise-intervention in older adults with knee osteoarthritis [46]. The J-MIND trial, aimed at preventing cognitive decline among older adults with Type 2 Diabetes and cognitive impairment in Japan by means of a multidomain intervention, found beneficial intervention effects on body weight [47]. It should be noted, however, that body weight was a pre-specified (secondary) outcome in this RCT, and intervention intensity was higher than in AgeWell.de, including, e.g., supervised physical activity and regular in-person nutritional counselling. Participants were slightly older on average and more cognitively impaired than in AgeWell.de.

Differences in body weight at follow-up were not associated with health-related, lifestyle- and psychosocial factors: The only covariates linked to body weight at follow-up were age (older participants having less body weight at follow-up), education (high levels of education being linked to lower body weight at follow-up) and body weight at baseline (higher values being linked to higher body weight at follow-up). Age-related differences in body composition may partly explain the observed association between higher age and lower body weight, as older adults generally have a reduced capacity to build muscle mass. However, the extent to which respective mechanisms may have influenced outcomes in the present study remains unclear. Although dementia was an exclusion criterion in the AgeWell.de-trial, we cannot rule out that preclinical cognitive or health-related changes contributed to unintentional weight loss in certain participants, which might explain the association between older age and lower body weight at follow-up observed in the present study. Neither were self-efficacy, motivation or confidence regarding weight loss associated with body weight at follow-up, nor did they influence between-group differences by group allocation.

Findings regarding the effects of education on weight loss in older adults are mixed, with some studies reporting no association of education with weight outcomes [48], others reporting better weight outcomes linked to higher levels of education [49], and further studies describing worse weight outcomes in participants with higher educational attainment [50]. Participants with high levels of education adhered better to the intervention components in AgeWell.de [51], which might explain the association of education with lower body weight observed in the current study.

In a previous study using the AgeWell.de data, beneficial intervention effects were detected on participants’ diet [34]. Consistent with these findings, supplementary analyses in the present subsample of AgeWell.de participants with overweight or obesity showed between-group difference in dietary quality at follow-up. One possible explanation refers to the assessment of a healthy diet in our study, which relied on a FFQ capturing consumption of foods and beverages using self-reports, without providing information on calorie intake. Despite improvements in diet quality, it might be that overall energy intake remained stable, or reductions in energy intake might have been insufficient to induce a sufficient reduction in body weight. However, it should be noted that dietary intake was self-reported in our study and the observed between-group difference was small, therefore, respective findings should be interpreted with caution. Higher age was linked to better adherence to a healthy diet at follow-up both in the previous analysis of the AgeWell.de-trial [34], and in the current post-hoc analysis. Although this finding might be related to the observed association between older age and lower body weight in the present sample, no data on total energy intake were available and information on participants’ diet was based on self-reports, therefore, this finding should not be interpreted as evidence for a causal effect of healthier eating on body weight in the present analysis. In line with this thought, nevertheless, Zhu and colleagues reported greater sustained weight loss in older than in middle-aged adults in a 3-year lifestyle intervention targeting weight loss in adults with prediabetes [52].

Physical activity was not linked to body weight at follow-up. Nevertheless, we detected higher physical activity scores at follow-up in the present post-hoc analysis, therefore, the absence of weight loss cannot be attributed to an absence of change in physical activity. More likely, increased activity levels may have been insufficient in frequency, intensity or duration to induce measurable weight loss. Assessment of physical activity in our study was based on a sum score comprising various self-reported activities with different intensities and durations, complicating estimations of actual energy expenditure and respective effects on body weight. Lastly, the intervention period was partly affected by the COVID-19 pandemic, with restrictions like lockdowns and social distancing limiting participants’ opportunities for physical activity.

Cardiometabolic diagnoses were not associated with body weight at follow-up in the total sample or participants with obesity, based on the primary multiple-imputation analysis. In sensitivity analyses using complete cases only, an association of cardiac arrhythmia with higher body weight was observed in participants with obesity. As this isolated association was not replicated in the primary analysis and arose from exploratory subgroup analyses without adjustment for multiple testing, it may represent a chance finding and should be interpreted with caution.

Evidence from dedicated weight-loss interventions differs from the present findings. For example, in the IDEAL-trial, motivation and self-efficacy were linked to adherence and actual weight loss, and the intervention also increased levels of these constructs [53]. However, the present study focused on cognitive performance and dementia risk, rather than weight loss. Although the intervention included components targeting diet and physical activity, weight management received comparatively limited emphasis. This may partly explain why motivation and confidence regarding weight loss did not emerge as predictors of body weight at follow-up. Future lifestyle interventions targeting older adults at risk for dementia aiming to influence body weight could therefore place greater emphasis on obesity-related information and explicitly link weight management to brain health and other modifiable risk factors for dementia. As overweight and obesity are associated with various cardiometabolic conditions, respective efforts might also be beneficial for risk reduction for other conditions such as hypertension, coronary heart disease or diabetes. Assessing further aspects like, e.g., body image, self-regulation skills or information on previous attempts to lose weight in RCTs investigating lifestyle interventions might provide further insights into psychosocial factors influencing weight loss in older adults.

Furthermore, levels of both motivation for weight loss and confidence in one’s ability to lose weight were not amendable to change due to the intervention and remained rather stable between baseline and follow-up. This might, at least partially, be explained by selection effects and limited variance, since all participants of AgeWell.de signed up for a relatively long, multidomain lifestyle intervention, suggesting high initial levels of motivation for behavior change. Indeed, 89.9% of participants in our sample of overweight and obese adults stated that losing weight was at least somewhat important to them, and 83.9% were at least somewhat confident that they could successfully reduce their weight (see Table 1). In a similar manner, self-efficacy was slightly skewed, with high average values at baseline (M: 22.6, SD: 4.8), observed range: 5–30), indicating potential ceiling effects, i.e., limited room for improvement regarding motivation and confidence concerning weight loss. Importantly, however, we detected favorable changes in both adherence to a healthy diet and physical activity in IG-participants with overweight or obesity. Therefore, the absence of weight loss in the present analysis should not be interpreted as a general failure to translate motivation into actual behavior change. Rather, it might be that the observed changes in diet and physical activity were insufficient in magnitude, duration or effect on energy balance to reduce body weight. This might, at least partially, reflect the fact that the AgeWell.de-intervention primarily targeted cognitive performance and dementia risk reduction rather than weight loss.

Generally, body weight might not be the most sensitive marker of beneficial lifestyle change in multidomain interventions targeting cognitive performance and dementia risk. These interventions might induce improvements in dietary quality, physical activity, vascular risk management or physical fitness, without substantial changes in total body weight. In AgeWell.de, for example, the intervention reduced the LIBRA-score, a validated index of modifiable dementia risk, mainly through improvements in diet and blood pressure [31], despite the absence of weight loss observed in the present analysis. Similarly, in the US MIND diet trial, the intervention improved adherence to the MIND diet and levels of several nutritional biomarkers (e.g., antioxidant levels), while no significant between-group differences were observed in body weight or cognitive performance after three years. [19]. This suggests that beneficial lifestyle changes may occur independently of measurable weight loss, i.e., stable body weight should not be interpreted as indicating an absence of broader health-related effects.

For interventions that explicitly aim to improve weight-related outcomes in older adults, greater intensity of weight-specific behavioral support might be required. This could include more frequent counselling or personal contacts during the intervention, repeated individualized action planning, self-monitoring, goal setting, and feedback on progress. The optimal frequency, duration, and combination of said components should be evaluated in future studies.

### Strengths and limitations

A strength of the present analysis is that it draws on objectively assessed data from a cluster-randomized trial, including a large set of covariates, and accounting for baseline body weight and clustering within GP practices. AgeWell.de followed the design of the multidomain FINGER-trial, and is part of the WW-FINGERS network, aiming to tailor the FINGER-approach to different regional, cultural and geographic conditions. Our findings might therefore aid the design and implementation of future interventions targeting risk factors for dementia in older at-risk samples. Due to the high prevalence of overweight and obesity and associated comorbidities in Western societies, our results have high relevance for both research and practice. A sound statistical approach taking into account baseline group differences in body weight and clustering of participants in GP practices further strengthen robustness of reported findings. Body weight was measured objectively, using a standardized anthropometric assessment conducted by trained study nurses, making our results robust against reporting bias.

Certain limitations should be acknowledged when interpreting our findings. Body weight was not a prespecified trial outcome, and the present analyses were conducted post hoc, therefore, the findings should be interpreted as exploratory. We aimed to keep burden for participants low, therefore, body weight was assessed at only two time points, i.e., baseline and follow-up after 24 months. This, however, prohibits statements about fluctuations in body weight and could mask transient or early changes in body weight, which were not sustained until the 24-month follow-up investigation. Assessments of physical activity and food intake were based on self-reports, complicating statements on actual changes in energy intake potentially related to body weight at follow-up. Since no information on whether weight loss was intentional or not, we cannot rule out the risk of reverse causation related to underlying disease or preclinical cognitive decline. The present subsample closely resembled the overall AgeWell.de baseline cohort reported previously with regard to sociodemographic characteristics and pre-existing conditions [29]. Nevertheless, because the current analysis was restricted post hoc to participants with overweight or obesity, the original balance between randomized groups cannot be assumed to have been fully preserved. Furthermore, the intervention period was partly affected by the COVID19-pandemic. While evidence from Germany suggests that (psychosocial) health consequences of pandemic restrictions like lockdowns and social distancing were generally low in older adults [54], the pandemic limited participants’ opportunities for conducting the intervention, e.g., regarding physical and social activity, which may have influenced our findings. Non-significant effects of, e.g., diet or physical activity might partially be explained by respective pandemic-related restrictions. Generalizability of our results is limited, as the intervention addressed older adults at risk for dementia. However, at-risk samples with a high number of comorbidities constitute an especially promising target group for lifestyle interventions, therefore, we are confident that our study still provides valuable insights for the design and implementation of future interventions in research and practice alike. Both motivation for weight loss and confidence in one’s ability to lose weight were measured using single items. More extensive assessments of said factors, or inclusion of further psychosocial measures like body image or self-regulation skills, may have provided more detailed information on what influences weight loss in older adults. This is underscored by studies showing that intrinsic, as opposed to extrinsic motivation, is particularly relevant in predicting weight loss due to lifestyle interventions [55].

## Conclusion

In this post-hoc analysis, the multidomain AgeWell.de intervention was not associated with lower body weight at follow-up. Although motivation and confidence regarding weight loss remained high and the intervention was associated with improvements in diet and physical activity, this did not translate into measurable weight loss in a multidomain lifestyle intervention focusing on cognitive health and dementia risk. Future interventions aiming to improve weight-related outcomes in older adults might therefore require a stronger and more explicit focus on weight management. Strengthening behavior change techniques such as self-monitoring, goal setting, and action planning might be particularly important for improving weight-related outcomes in older adults at risk for dementia.

## Supplementary Information

Below is the link to the electronic supplementary material.

Supplementary Material 1

## Data Availability

The dataset analyzed during this study are not publicly available due to privacy restrictions. However, data are available after de-identification to researchers who submit a sound proposal to the AgeWell.de steering committee. Requests to access the dataset should be submitted to: Steffi.Riedel-Heller@medizin.uni-leipzig.de.
