# Supplementary material for: Body weight following a multidomain lifestyle intervention in older adults at risk of dementia – A post-hoc analysis of the AgeWell.de-trial
Source: BMC Geriatr. 2026 Oct 15;26:1298. doi: 10.1186/s12877-026-08492-6 (PMC13673888; doi:10.1186/s12877-026-08492-6)
Supplement: Supplementary file 1 [file 12877_2026_8492_MOESM1_ESM.docx]

**Supplementary Material**

**Zülke et al., Body weight following a multidomain lifestyle intervention in older adults at risk of dementia – A post-hoc analysis of the AgeWell.de-trial**

**Supplementary Table 1: Adjusted associations with body weight at 24-month follow-up among participants with BMI ≥25 kg/m², including interactions of group allocation and self-efficacy, motivation for and confidence regarding weight loss, imputed data (BMI ≥ 25 kg/m²; n = 678)**

| **Variable** | **b**  **(b)** | **95% CI** | ***p*** |
| --- | --- | --- | --- |
| Intervention group (ref.: control group) | -0.92 | -4.94; 3.10 | .650 |
| Body weight at baseline | 0.95 | 0.91; 0.98 | **<.001** |
| Age (years) | -0.14 | -0.25; -0.03 | **.014** |
| Female sex (ref.: male) | -0.86 | -1.82; 0.10 | .077 |
| Married/relationship (ref.: single) | -0.72 | -1.64; 0.19 | .120 |
| Education level intermediate (ref.: low) | 0.03 | -1.08; 1.14 | .960 |
| Education level high (ref.: low) | -1.29 | -2.56; -0.02 | **.047** |
| Self-efficacy (GSE-score) | -0.05 | -0.17; 0.07 | .424 |
| Motivation for weight loss | -0.22 | -0.76; 0.32 | .424 |
| Confidence in ability for weight loss | 0.23 | -0.19; 0.65 | .281 |
| IG*self-efficacy | 0.00 | -0.18; 0.18 | .998 |
| IG*motivation for weight loss | 0.43 | -0.28; 1.13 | .235 |
| IG*confidence in ability for weight loss | -0.13 | -0.81; 0.56 | .713 |
| Smoking (ref.: non-smoker) | -0.17 | -1.41; 1.06 | .781 |
| Physical activity, total score | 0.01 | -0.08; 0.09 | .874 |
| Healthy diet, total score | 0.01 | -0.24; 0.26 | .931 |
| Diabetes mellitus | -0.47 | -1.12; 0.19 | .159 |
| Hypertension | -0.34 | -1.38; 0.71 | .524 |
| Hyperlipidemia | -0.38 | -1.28; 0.53 | .409 |
| Cardiac insufficiency | -0.97 | -2.66; 0.72 | .256 |
| Cardiac arrhythmia | 0.33 | -0.77; 1.44 | .553 |

b: coefficient; BMI: body mass index; CI: confidence interval; GSE: Generalized Self-Efficacy Scale; IG: intervention group; ref.: reference

**Supplementary Table 2: Adjusted associations with body weight at 24-month follow-up among participants with BMI ≥25 kg/m², complete case analysis (BMI ≥ 25 kg/m²; n = 557)**

| **Variable** | **b**  **(b)** | **95% CI** | ***p*** |
| --- | --- | --- | --- |
| Intervention group (ref.: control group) | -0.21 | -1.13; 0.71 | .648 |
| Bodyweight at baseline | 0.94 | 0.90; 0.98 | **<.001** |
| Age (years) | -0.21 | -0.36; -0.08 | **.002** |
| Female sex (ref.: male) | -0.93 | -2.05; 0.19 | .102 |
| Married/relationship (ref.: single) | -1.17 | -2.12; -0.21 | **.018** |
| Education level intermediate (ref.: low) | -0.39 | -1.60; 0.83 | .528 |
| Education level high (ref.: low) | -2.13 | -3.60; -0.65 | **.005** |
| Self-efficacy (GSE-score) | -0.32 | -1.22; 0.58 | .483 |
| Motivation for weight loss | -0.06 | -0.51; 0.38 | .775 |
| Confidence in ability for weight loss | -0.02 | -0.45; 0.41 | .944 |
| Smoking (ref.: non-smoker) | -0.61 | -2.04; 0.81 | .395 |
| Physical activity, total score | 0.24 | -0.78; 1.27 | .640 |
| Healthy diet, total score | -0.06 | -0.35; 0.22 | .670 |
| Diabetes mellitus | -0.15 | -0.91; 0.60 | .687 |
| Hypertension | -0.63 | -1.99; 0.72 | .354 |
| Hyperlipidemia | -0.50 | -1.44; 0.45 | .301 |
| Cardiac insufficiency | -1.55 | -3.55; 0.45 | .127 |
| Cardiac arrhythmia | 0.96 | -0.31; 2.24 | .137 |

b: coefficient; BMI: body mass index; CI: confidence interval; GSE: Generalized Self-Efficacy Scale; ref.: reference

**Supplementary Table 3: Adjusted associations with body weight at 24-month follow-up among participants with obesity (BMI ≥ 30 kg/m²; n = 386), complete case analysis**

| **Variable** | **b** | **95% CI** | ***p*** |
| --- | --- | --- | --- |
|  |  |  |  |
| Intervention group (ref.: control group) | -0.15 | -1.36; 1.06 | .804 |
| Bodyweight at baseline | 0.93 | 0.86; 0.99 | **<.001** |
| Age | -0.16 | -0.33; 0.01 | .059 |
| Female sex (ref.: male) | -1.16 | -2.69; 0.36 | .134 |
| Married/relationship (ref.: single) | -1.09 | -2.35; 0.16 | .086 |
| Education level intermediate (ref.: low) | -0.16 | -1.76; 1.45 | .846 |
| Education level high (ref.: low) | -1.58 | -3.58; 0.42 | .119 |
| Self-efficacy (GSE-score) | -0.53 | -1-69; 0.62 | .363 |
| Motivation for weight loss | 0.07 | -0.54; 0.67 | .831 |
| Confidence in ability for weight loss | 0.07 | -0.46; 0.61 | .781 |
| Smoking (ref.: non-smoker) | 0.06 | -1.86; 1.97 | .955 |
| Physical activity, total score | 0.02 | -1.36; 1.39 | .981 |
| Healthy diet, total score | -0.08 | -0.44; 0.28 | .677 |
| Diabetes mellitus | -0.12 | -1.09; 0.85 | .808 |
| Hypertension | -1.08 | -2.84; 0.69 | .228 |
| Hyperlipidemia | -0.39 | -1.60; 0.82 | .525 |
| Cardiac insufficiency | -1.82 | -4.42; 0.79 | .169 |
| Cardiac arrhythmia | 1.81 | 0.10; 3.52 | **.039** |

b: coefficient; CI: confidence interval; GSE: Generalized Self-Efficacy Scale; ref.: reference

**Supplementary Table 4: Intervention effects on behavioral outcomes at follow-up (n = 678, imputed data)**

|  | **PA, total score** | | | **Healthy diet, total score** | | |
| --- | --- | --- | --- | --- | --- | --- |
| **Variable** | **b** | **95% CI** | **p** | **b** | **95% CI** | **p** |
| Intervention group (ref.: control group) | 2.22 | 1.56; 2.87 | **<.001** | 0.24 | 0.01; 0.48 | **.039** |
| Baseline value of PA /healthy diet-score | 0.59 | 0.52; 0.66 | **<.001** | 0.54 | 0.46; 0.61 | **<.001** |
| Age | -0.07 | -0.12; -0.01 | **.019** | 0.03 | 0.46; 0.61 | **<.001** |
| Female sex (ref.: male) | 0.69 | 0.10; 1.27 | **.022** | 0.34 | 0.11; 0.57 | **.004** |
| Married/relationship (ref.: single) | 0.49 | -0.27; 1.25 | .205 | 0.03 | -0.27; 0.34 | .819 |
| Education level intermediate (ref.: low) | 0.20 | -0.60; 1.00 | .615 | -0.16 | -0.46; 0.14 | .292 |
| Education level high (ref.: low) | 1.57 | 0.76; 2.38 | **<.001** | -0.15 | -0.47; 0.18 | .369 |

Linear regression models assessing effects of intervention group allocation (ref.: control group) on physical activity and a healthy diet, higher scores indicating better adherence. Cluster-robust standard errors accounted for clustering within GP practices. Estimates were pooled across 20 imputed datasets. b: coefficient; CI: confidence interval; PA: physical activity; ref.: reference
